# Managing host-parasite interactions in humans and wildlife in times of global change

**DOI:** 10.1007/s00436-022-07649-7

**Published:** 2022-09-06

**Authors:** Konstans Wells, Robin Flynn

**Affiliations:** 1 Department of Biosciences, Swansea University, Swansea, SA28PP UK; 2 Graduate Studies Office, South East Technological University, Cork Road Campus, Waterford, X91 K0EK Ireland

**Keywords:** Parasite control, Parasitic networks, Eco-epidemiological dynamics, Paradox of increased global health, Outbreak control, Host-parasite system dynamics

## Abstract

Global change in the Anthropocene has modified the environment of almost any species on earth, be it through climate change, habitat modifications, pollution, human intervention in the form of mass drug administration (MDA), or vaccination. This can have far-reaching consequences on all organisational levels of life, including eco-physiological stress at the cell and organism level, individual fitness and behaviour, population viability, species interactions and biodiversity. Host-parasite interactions often require highly adapted strategies by the parasite to survive and reproduce within the host environment and ensure efficient transmission among hosts. Yet, our understanding of the system-level outcomes of the intricate interplay of within host survival and among host parasite spread is in its infancy. We shed light on how global change affects host-parasite interactions at different organisational levels and address challenges and opportunities to work towards better-informed management of parasite control. We argue that global change affects host-parasite interactions in wildlife inhabiting natural environments rather differently than in humans and invasive species that benefit from anthropogenic environments as habitat and more deliberate rather than erratic exposure to therapeutic drugs and other control efforts.

## Introduction

Parasites are estimated to comprise at least half of all living species (Larsen et al. 2017), evidencing the success of life history strategies that involve invading other species as a habitat. Their successful proliferation and spread often impairs host fitness and behaviour by co-opting host resources for their own survival and reproduction and disrupting the hosts’ homeostasis (Horak et al. 2004). They can fundamentally impact host health, population dynamics and extinction risk, with potentially far-reaching consequences of how host species can realise their functional role in providing ecosystem services (Gagne et al. 2022). Parasites can also modulate their host’s interactions with other organisms and affect entire food web dynamics (Lafferty et al. 2008). Host species in turn, possess an array of defence strategies to resist infection and parasite growth, ranging from behavioural avoidance to mounting protective immune responses. Ultimately, successful parasite proliferation requires eco-evolutionary stable host-parasite relationships that continuously ensure efficient growth, survival and reproduction within host individuals as well as dispersal among different host individuals for continuous persistence of the parasite.

The large-scale pandemic spread of SARS-CoV-2 through human populations has resulted in an unprecedented amount of novel data and research endeavour, embracing aspects as diverse as pathology, epidemiology, pathogen phylodynamics, socioecological and environmental conditions as drivers of pathogen spread and the efficacy of intervention strategies (Cevik et al. 2021; Edwards et al. 2022; Kraemer et al. 2020; Peeling et al. 2022). The zoonotic origin of SARS-CoV-2 has also reinvigorated interest in potential global change effects on infectious disease emergence and the origin of zoonotic parasites.

In fact, almost all species on earth, are likely to be affected by human activity, be it through climate change, habitat modifications, biotic invasions, pollution or human intervention in the form of mass drug administration (MDA) or vaccination.

Possible consequences are likely to affect parasites as much as non-parasitic organisms at the various organisational levels of life, encompassing eco-physiological stress at cell and organism level, individual fitness and behaviour, population viability, biotic interactions and food web dynamics. Yet, for the majority of studied parasites, particularly those infecting wildlife only, the impact of global change on host-parasite interactions remain poorly understood. Moreover, our current empirical understanding of host-parasite interactions still arises predominantly from ‘static’ perspectives that do not account for the synergistic effects of within and among host-parasite interactions (Handel and Rohani 2015) and possible spatiotemporal heterogeneity in environmental conditions that may alter how the interaction strength between hosts and parasites differ in space and time. By simplifying assumptions and disconnecting views of within and among hosts interactions, we may inadvertently conceal aspects of the underlying biology driving parasite spread throughout possible networks of interacting host and parasite organisms and the system-level dynamics arising from these interactions.

In this paper, we shed light on how global change affects host-parasite interactions at different organisational levels, identifying challenges and opportunities to work towards a more informed parasite management approach that attempts to anticipate the eco-epidemiological dynamics underlying host-parasite interactions. We begin by reviewing recognised global change phenomena to alter host-parasite interactions at different organisational levels. We then discuss possible differences in global change drivers and how these may synergise in their impact on host-parasite interactions in humans and domestic animal species versus wildlife before finally considering challenges and opportunities to work towards a more holistic evidence-base for parasite control.

Here, we use the terms ‘parasite’ and ‘pathogen’ often interchangeably, as we believe that most of our arguments around systems-level approaches are relevant for both groups. We argue that a more thorough understanding of the intricate interplay of within host survival and among host parasite spread in the context of environmental heterogeneity and stressors will improve our ability to forecast host-parasite interactions and implement more efficient control strategies in light of ongoing global change.

### Host-parasite interactions in the Anthropocene

The Anthropocene has heralded changes in public health measures and actions to improve food safety (Whitmee et al. 2015). These have had immense positive impact on the morbidity and mortality of human populations and these benefits extend to domestic animal health and welfare. The general outcome is that humans are living longer lives with improved access to more and better quality food, health care and hygiene. These benefits have arisen from the progress made in veterinary and human medicine, including advancements in diagnostic capacity (Djuardi et al. 2022), anti-parasitic drugs (Tavul et al. 2022), vaccines (Lightowlers et al. 2021), and, more recently, bioengineering approaches to neutralise disease vectors (Wang et al. 2021; Windbichler et al. 2011). Conversely, this period of development has in parallel experienced ongoing negative changes to the natural environment in response to an ever-increasing demand of natural resources and developed space (Crippa et al. 2021). In particular, the increased demands placed upon the global food supply has resulted in expansion of land utilised for agricultural purposes and intensification of homogenous agricultural practices, including considerable shifts in biomass from wildlife to farmed and domestic animals (Bar-On et al. 2018). This has resulted in a parallel degeneration and fragmentation of natural habitats, exhilaration of wild species extinction rates and invasive species spread (Díaz et al. 2019). These downstream consequences are likely to affect host-parasite interactions and thus the efficacy of parasite control in manifold ways. This same paradox can be also applied to the current challenge to tackle antimicrobial resistance as a major threat to food security: successful treatment of bacterial infections with antibiotics may contain bacterial infections within focal host individuals, while potentially imposing increasing risk of antimicrobial resistance within populations and across species (Ahlstrom et al. 2021; Bengtsson-Palme et al. 2018; Naylor et al. 2018).

Arguably, we are now entering a period where these multiple impacts are merging to produce novel conditions for parasite spread and challenges for parasite control. Mounting evidence suggests that outbreaks of emerging infectious disease are becoming more frequent through global change events that spark them (Franklinos et al. 2019; Karesh et al. 2012). Globalisation and increasing connectivity of human and domestic animal populations at global scale has facilitated unprecedented species invasions (Dawson et al. 2017). Moreover, land use change, habitat fragmentation and the diminishing boundaries between natural and anthropogenic environments means that contact opportunities between humans and domestic animal with formerly isolated wildlife species can intensify and facilitate shifts of parasites into novel hosts and spillover events (Morand 2020; Plowright et al. 2021; Wells et al. 2018).

From a broader ecological perspective, it is crucial to consider that essentially almost all species’ occurrence, abundance and biotic interactions can be considerably altered by global change. Climate and land use change and species invasions all have been widely documented to impact the ecophysiology, phenology, population dynamics and distribution of species worldwide (Essl et al. 2020; Parmesan and Yohe 2003; Pörtner and Farrell 2008). In the case of tick-borne diseases, for example, changes in abiotic condition such as temperature and humidity can alter the physiology of individual organisms such as acarid ticks that require suitable climate conditions to ambush for their vertebrate host (Randolph 2001). At broader geographic scale, there is clear evidence that the northern range margin of pathogen-transmitting *Ixodes* spp. ticks is expanding in response to climate change in the northern hemisphere (Ogden et al. 2020). This evidence coupled with seroprevalence data for *Babesia* spp. and other tick-borne pathogens, would suggest that increased prevalence of these pathogens may well be a direct result of the expanding range of vectors due to climatic change (Springer et al. 2020). Critically, the formation of local interactions between ticks and their host within a suitable habitat can depend on the outcome of local environmental conditions and biodiversity; therefore, the capacity for parasites to spread depend on entire food web dynamics of predator, prey and reservoir host species (Ostfeld et al. 2006).

**Fig. 1:**
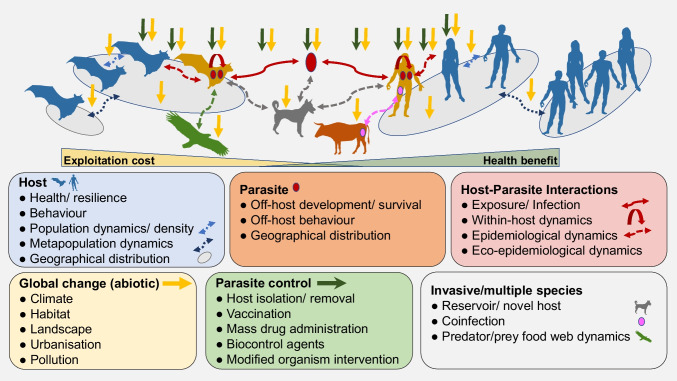
Conceptual illustration of host-parasite interactions at different organisational levels with possible impact of global change, parasite control and invasive species. We aimed to list key stages of host-parasite interactions with increasing complexity from single host exposure to complex eco-epidemiological dynamics. Although global change and parasite control impact are multifaceted, we postulate here that parasite control most focuses at the levels of host exposure and within-host dynamics, whereas global change can impact virtually any species or interaction formation within entire biological systems. If the ultimate aim of parasite control effort is the reduced impact of the parasite on the host at higher organisational level (i.e. populations and species), the emergent system dynamics arising from all factors may determine the efficacy of any control measure in variable environments. The relative size difference in the illustrative human versus wildlife host populations demonstrates the idea that humans may disproportionately benefit from health improvements and parasite control in comparison to wildlife that suffer the most from resource exploitation

Since global change can impact virtually any species or interaction formation within biological systems, the outcome of parasite control efforts may not only depend on the control method per se but, rather, its application in the given ecological and spatiotemporal context. Therefore, if the ultimate aim is the long-term and broad-scale control of a parasite, selecting the most appropriate management strategy could benefit from taking system-level dynamics arising from biotic interactions among multiple species and the effects of global change and management into account (Fig. 1).

To this end, we have only mentioned key aspects of global change and possible impacts on host-parasite interactions at different organisational levels. While it is obvious that global change has far-reaching consequences on the mechanisms of species occurrence and interactions and therefore the spread and impact of parasites, we are only in its infancy to understand the multifaceted way global change can impact host-parasite interactions. The paradox of improved health and natural system deterioration (Whitmee et al. 2015) is just one example of contradictory short-term versus long-term benefits, while we may also expect that parasite control efforts that work well under constant conditions are not necessarily the best option in variable environments.

Addressing these global challenges in parasite control has resulted in multiple calls for ecosystem-level approaches. The Word Health Organisation (WHO) has recognised more than a decade ago that sound infectious diseases risk prediction and parasite control is based upon improved frameworks of information exchange at the human-animal-ecosystems intersections, thus, highlighting the need for a greater understanding of possible mechanisms and processes shaping host-parasite interactions within the ‘OneHealth’ and ‘Planetary Health’ frameworks (Whitmee et al. 2015). More recently, unprecedented outbreaks, such as Covid-19, Ebola, and Zika, have put renewed emphasis on prevention rather than reaction to the spread of infectious diseases among humans and animals (Morens and Fauci 2020). This calls for holistic and ecosystem-based approaches for understanding the ecology and epidemiology of what brings about some parasites to develop virulent transmission dynamics and spillover into novel host species in comparison to the myriads of benign microorganisms and pre-emergent pathogens. Such ecosystem-level thinking can be equally helpful for understanding and managing parasite spread in wildlife populations (Hassell et al. 2021). However, we must bear in mind that the health paradox of increased global health at the cost of overexploitation of natural resources means that global change phenomena can constantly impact the success of parasite control strategies. If improving the health and infection status for one host population or species disadvantages other host populations or species or results in spatiotemporally shifted resource depletion, knowledge gaps remain and such shortfalls could guide hypotheses to be addressed in future research.

### Host-parasite interactions in humans and domestic animals versus wildlife

The paradox of improved health concept can be also a starting point for asking of whether host-parasite interactions in humans and domestic animals are differently impacted by global change than those in wildlife and whether such differences are relevant for parasite control strategies.

Much focus in recent years have been placed on the pathogen emergence process in context of possible changing human-domestic animal-wildlife interfaces driven by landscape modifications, climate change and other global change events that alter contact opportunities between humans and animal reservoirs and may enable host shifting and parasite spillover (Carlson et al. 2022; Gibb et al. 2020; Glennon et al. 2021; Wells et al. 2020). For wildlife, rapidly expanding international trade potentially results in more frequent contact opportunities among humans and wildlife and therefore exposure to different parasites (Pavlin et al. 2009). Thus, a growing number of novel contact opportunities among different species may result in host shifting and an ever-growing number of associated parasite species for both humans and wildlife species alike. However, demographic changes in human and domestic species versus wildlife can be rather different in times of global change. The large-scale dimensions of pandemic outbreaks in humans, for example, have demonstrated that the growing global connectivity of human populations, including human migration, international travel and local-scale mobility along with changeable human behaviour, inexorably facilitates parasite spread in human populations (Baker et al. 2021; Findlater and Bogoch 2018; Heesterbeek et al. 2015). In contrast, for many wildlife species, landscape modifications largely result in reduction and fragmentation of natural habitats. Consequently, for many wildlife species, global change is most likely to reduce species population density and also population connectivity. Especially in endangered wildlife species, smaller population sizes and low connectivity of populations may reduce the spread of parasites at metapopulations level, while it may also disrupt host colonisation dynamics and gene flow critical for maintaining healthy populations (Durrant et al. 2021; Jousimo et al. 2014). Other ways of impacting wildlife populations such as hunting have recently also been suggested to alter the viral transmission dynamics and phylogenetic diversity in a large carnivore species (Fountain-Jones et al. 2022). For multi-host parasites capable of infecting both domestic and wildlife species, reduced wildlife host availability and shift towards predominantly peridomestic species in anthropogenic environments can cause altered transmission dynamics. Shift from sylvatic to domestic transmission cycles, for example, have been proposed to alter the pathogen’s virulence for the protozoan parasite *Toxoplasma gondii* (Shwab et al. 2018) and likely contributed to the unexpectedly large-scale intercontinental spread of the swine fever virus among domestic and wild pigs (Dixon et al. 2020). Another potential key difference between human, domestic species and wildlife species is the exposure history and diversity of associated parasites, which may determine levels of cross-immunity and resistance when exposed to novel pathogens. Animal species with the longest domestication history, for example, share most parasite with humans (Morand et al. 2014) and for parasites with a long history of being associated with a host species or closely related parasites, potential cross-immunity could affect host-parasite interactions and epidemiological dynamics (McCormick et al. 2021). Wildlife species with limited pre-exposure to certain parasites, in turn, typically suffer from the most severe outbreak and host extinction risk (Lips 2016; Tompkins and Jakob-Hoff 2011). We may expect some differences in host-parasite interactions between domestic and wildlife species to become more extreme; the more wildlife species populations are shrinking or threatened (Díaz et al. 2019; Faust et al. 2018) and the more the populations and biomass shifts from wildlife to domestic animals and humans (Bar-On et al. 2018). At the same time, increasing biotic homogenisation and the long history of hundreds to thousands of years of contact of humans and domestic animals with the majority of wildlife species questions whether novel contact opportunities should indeed increase or rather become less important over time compared to other global change features as drivers of host-parasite interactions.

Potential differences in host-parasite interactions in humans and domestic animals versus wildlife could also affect parasite control efforts. Perhaps some lessons have been learnt from parasites that affect both groups of host species. Interventions for the benefit of human health can be more targeted and better controlled when we consider the domestic animal-human interaction, resulting in greater success. The pig tapeworm, *Taenia solium*, for example, causes cysticercosis in the porcine host but when transmitted to humans result in neurocysticercosis giving rise to high morbidity neurological disease. While vaccination to control infection in the porcine host is both logistically possible and gives rise to protection, it is mostly implemented due to the indirect effect on human disease. Incorporation of a targeted MDA element in these vaccination campaigns and understanding the spatial distribution of all possible hosts are increasingly considered in roadmaps for control (Conlan et al. 2011); CystiTeam Group, 2019). Reliance on a single approach to control, while tempting, is fraught with potential problems. MDA and the emergence of drug resistant parasites represent a prime example of the speed with which positive human interventions can be rendered meaningless. The time from introduction of a new anti-parasitic drug to emergence of resistance can in some cases be very short (Bartley et al. 2015; Davies and Davies 2010). However, possible trade-offs in the face of declining drug efficacy due to the limited availability of alternative control methods or the need for continuous parasite control can have implications for how we approach drug usage. These decisions can only be informed by a more detailed understanding of the parasite within the context of its ecosystem, i.e. the manner in which host population structures and host resistance drive parasite spread and make parasite control feasible beyond a limited range of targeted host individuals. Moving beyond this, incorporation of systematic monitoring and understanding the movement of drug resistant genomes within the parasite population (Ndiaye et al. 2021) and the invasion success of drug resistant parasite in host microbial and parasite assemblages (Letten et al. 2021) will be key for informed drug use in parasite control. The advent of affordable and fast in-field genomic technologies makes this approach more feasible.

Summarising these aspects, we see several research frontiers as offering the potential to better understand aspects of disease management in humans and domestic animals versus wildlife:

(i) Gain a better understanding of how managing host-parasite interactions in focal populations and species, especially humans and domestic animals, results in unwanted health impairments in other populations and species such as through the uncontrolled spread of antimicrobial resistant parasites.
  Here, omics-based monitoring of the effects of released drugs on focal and non-focal host populations combined with cost benefit analysis of different drug release schemes could be useful tools. The application of environmental DNA (eDNA) approaches, used widely in environmental impact studies, can identify large scale community changes through the incorporation of a metabarcoding step (Djurhuus et al. 2020).
(ii) Gain a better understanding of how host-parasite interactions in humans and domestic animals differ from those in wildlife inhabiting natural environments with less dense and connected populations and less intense exposure history to parasites.
  Here, comparative, empirical studies of parasite load, transmission, and distribution combined with studies of host immunological resistance in human and animal populations can be used to generate data for system-dynamical computer simulation. The growing capacity for systems-level immunological profiling from decreasing sample volumes makes this approach feasible for humans and a growing consideration for other species when coupled with novel CyTOF mass cytometry labelling approaches (Olin et al. 2018).
(iii) Develop and refine models of multi-host host-parasite interactions and control strategies that account for multiple host species at human-domestic animal-wildlife interfaces embedded within variable environments.
  Here, system-dynamical computer simulations that account for multiple species interactions while modelling the outcome of various control scenarios could be beneficial. Data obtained from empirical studies and environmental monitoring would serve to train and build these models.
(iv) Gain a better understanding of how environmental stressors may impact pathogen transmission dynamics and evolution in context of possible individual heterogeneity in host susceptibility and tolerance to infections.
  Here, field-based and experimental studies that measure host resilience and tolerance in response to various environmental stressors such as heat, drought, pollution, coinfection and interspecific competition could be of interest.

### A system-dynamical perspective towards better-informed parasite control

Anticipating that parasite spread involves spatiotemporal processes and interactions of at least two but often many more species, we believe that decision makers should critically scrutinise the efficacy of parasite control strategies, while minimising possible unwanted side effects at various organisational levels.

**Fig. 2:**
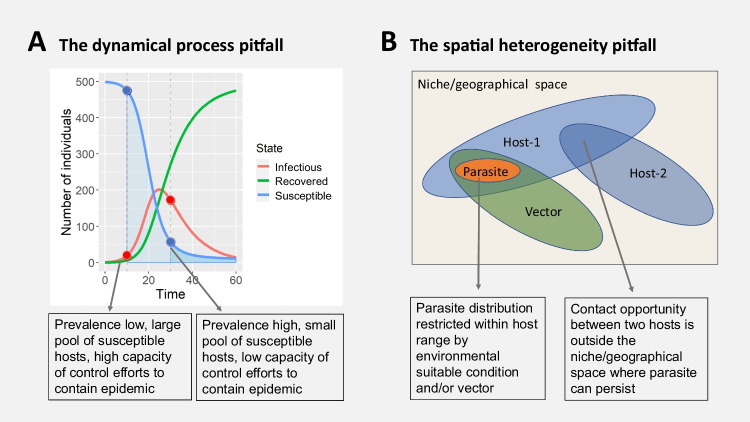
Possible pitfalls in concluding on risk of parasite spread and spillover from epidemiological dynamics and spatial heterogeneity in species interactions. Panel **A** illustrates a possible dynamical process pitfall: if parasite control decisions are guided by prevalence only, focusing control efforts to populations with high prevalence may result in less overall containment of parasite spread if the pool of susceptible individuals is low towards a later stage of an endemic (dark-blue shaded area), whereas containing parasite spread in a population with low prevalence but large pools of susceptible hosts may protect a larger proportion of a population from infection (light-blue shaded area) and lower the force of infection. Panel **B** illustrates a possible spatial heterogeneity pitfall: parasites can be confined to small subsets of a host’s niche/geographical space if environmental conditions do not allow to maintain transmission dynamics throughout a host’s range or if essential vector species are not present. Hence, the area of pathogen presence and spillover can be overestimated when host-parasite formation are assumed to take place throughout the niche/geographical space of a host species. In such a scenario, contact opportunities between different host species do not necessarily allow to conclude on pathogen spillover

Exhaustive surveillance of parasite spread in space and time is often hindered by lack of resources and feasibility (Tambo et al. 2014), meaning that sufficient data for understanding parasite spreading processes in space and time are not available for a large number of parasites. Possible challenges may include that ‘static’ snapshot observations such as parasite incidence records from a limited spatiotemporal window allow limited insights into the underlying dynamical processes. Moreover, conclusions drawn from initial epidemics may be biased by transient dynamics that allow limited insights into long-term disease spread and impact on host populations (Hastings 2004; Wells et al. 2019). Informed parasite control should be guided by model-based forecasting, taking advantage of the continuous development of multivariate modelling frameworks that allow users to account for dynamical and multiscale processes for improved predictions of the spatiotemporal plasticity in host-parasite interactions (Clark and Wells in press; Simonis et al. 2021; Zipkin et al. 2021). Anticipating that the spread of any parasite depends on underlying connectivity networks, plasticity of host connectivity should be also considered when comparing key epidemiolocal parameters such as the basic reproductive number *R*_0_ over space and time and among different species. Scientific conclusions and informed parasite control efforts should ideally strike a right balance between practical decision making and transparent rationales of how the dynamics and plasticity in host-parasite interactions can be taken into account. The practical decision of controlling a parasite at a ‘hotspot’ of high prevalence can be less efficient in containing overall parasite spread than controlling the same parasite at a site with low prevalence but larger pool of susceptible individuals early in an epidemic (Fig. 2). Single data streams, such as the number of infected individuals in the absence of knowledge of the proportion of immune individuals, can create a dynamical process pitfall in that decision makers are convinced they manage populations at high risk, while the real-world outcome can be worse in terms of overall parasite spread if the underlying processes and dynamics are ignored (Fig. 2).

Likewise, simply projecting host-parasite associations over entire species range distributions without accounting for the plasticity in a host-parasite association may misdirect scientists and policy makers to the wrong location of where and among which species future pathogen spillover may occur. Such simplified projections may overestimate host-parasite interaction formation and can create a spatial heterogeneity pitfall in that decision makers are convinced host range can predict pathogen spillover and outbreak risks, while the real risk may be confined to areas with suitable environmental conditions and species assemblages (i.e. parasite can be confined to those parts of a host range where environmental conditions allow maintained transmission dynamics and where suitable vectors are present) (Fig. 2). Good practices of reporting uncertainty and scrutinising assumptions in model-based forecasting and risk assessment is a good way forward to help decision makers in parasite control to avoid such pitfalls. Where key parameters of host-parasite interaction dynamics cannot be directly inferred from empirical data, computer simulations may aid in better predicting host-parasite interactions and the outcome of parasite control interventions (Drake et al. 2015). Such simulations require careful considerations of model structure and sufficient evidence from empirical data (Oberpriller et al. 2021), whereby the increasing availability of multiple data streams on host-parasite interactions will aid in informing such models. A better understanding of the immune response within human and animal hosts, for example, could help inform ongoing vaccine designs. At the same time, the success of vaccination and other parasite containment strategies can be optimised if accounting for population structure and dynamics (Britton et al. 2020) and the ‘landscape’ of host susceptibility and immunity (Becker et al. 2020). Crucially, the spread of any parasite within host populations is a dynamic process in space and time. Advances in rapid parasite surveillance and tracing of parasite spread through molecular sequencing and phylodynamic modelling have much improved our understanding of the spatiotemporal dimension of host-parasite interactions (Kraemer et al. 2019). Recent advances in sequencing-based (genomics/metagenomics, transcriptomics, epigenomics) and spectrometry-based (proteomics, lipidomics, and metabolomics) approaches may further improve our understanding of the physiological state, coinfections and microbiomes of host individuals prior and after infection, informing eco-epidemiological models of host resistance and parasite spread (Papaiakovou et al. 2022; Wanelik et al. 2020) (van Leeuwen et al. 2019).

## Conclusions

We have argued that virtually all of the mechanisms underlying host-parasite interactions and operating at different organisational levels and spatiotemporal scales can be altered by global change. From a system-level perspective, parasite control measures can be reduced to another factor in host-parasite interaction dynamics. Therefore, a more thorough understanding of the intricate interplay of within and among host parasite spread in the context of environmental heterogeneity and stressors will improve our ability of model-based forecasting of host-parasite interactions and the success of parasite control strategies. Furthermore, increasingly sophisticated parasite control strategies and more rigorous and detailed ecoepidemiological studies promise to provide not only insights into the efficacy of control strategies per se, but also into long-term and large-scale consequences of parasite control in times of global change.
